# Supplementary material for: Enhanced Cardiac CaMKII Oxidation and CaMKII-Dependent SR Ca Leak in Patients with Sleep-Disordered Breathing
Source: Antioxidants (Basel). 2022 Feb 8;11(2):331. doi: 10.3390/antiox11020331 (PMC8868143; doi:10.3390/antiox11020331)
Supplement: Supplementary file 1 [file antioxidants-11-00331-s001.zip › antioxidants-1516699-supplementary.pdf]

# Online Supplement

# Enhanced CaMKII-dependent SR Ca leak in atrial cardiomyocytes of patients with sleep-disordered breathing

Michael Arzt*; Marzena A. Drzymalski*; Sarah Ripfel; Sebastian Meindl; Alexander Biedermann; Melanie Durczok; Karoline Keller; Julian Mustroph; Sylvia Katz; Maria Tafelmeier; Simon Lebek; Bernhard Flörchinger; Daniele Camboni; Sigrid Wittmann; Johannes Backs; Christof Schmid; Lars S. Maier; Stefan Wagner

## Supplemental detailed methods

### Study design

Consecutive patients undergoing elective coronary artery bypass graft (CABG) were prospectively included after informed consent (fig. 1). Right-atrial appendage biopsies were sampled from one randomly selected patient per working day, who underwent CABG surgery. Assessment of SDB in clinical routine in the night before surgery was an inclusion criterion. Exclusion criteria were known sleep apnea and Continuous Positive Airway Pressure (CPAP)-therapy. Functional measurements of atrial cardiac myocytes and protein analysis of atrial biopsies were performed by blinded investigators.

### Assessment of SDB

Nasal flow, pulse oximetry and thoracal breathing effort were measured using the ApneaLink device (ResMed, Sydney, Australia) that has been validated in several studies for screening of SDB [1-6]. Comparing ApneaLink (using automatic scoring) to the gold standard polysomnography (PSG) in patients without known heart disease, studies have reported a sensitivity of 73-94% and a specificity of 85-95% using an AHI cut-off value of 15/h [7]. As prescribed previously [7] the default settings of the screening device were used for the definitions of apnea, hypopnea and desaturation: apnea was defined as a ≥80% decrease in airflow for ≥10 seconds; hypopnea was defined as a decrease in airflow by ≥50-80% versus baseline for ≥10 seconds; desaturation was defined as a ≥4% decrease in oxygen saturation; SDB was defined as AHI ≥15/h.

### Human Samples

Right atrial (RA) appendage biopsies were received from patients during elective coronary artery bypass grafting, after they had given written informed consent. Biopsies were transported to our laboratory in ice-cold Custodiol solution (Dr. Franz Köhler Chemie), containing (mmol/L): 15 NaCl, 9 KCl, 4 $MgCl_2$ x 6 $H_20$, 18 L-Histidinhyrochlorid x $H_20$, 180 L-Histitin, 2 Tryptophan, 30 Mannitol, 1 2-Oxoglutarsäure, 2 mmol/L butanedione monoxime, 0.015 $CaCl_2$ x 6 $H_20$. All experiments were approved by the local ethics committees and are in accordance with the Helsinki Declaration of 1975, as revised in 2008.

### Isolation of human atrial cardiomyocytes

RA biopsies were used for chunk isolation as previously described[8] with sliced tissue incubated at 37°C in a spinner flask filled with Tyrode solution (in mmol/L 88 NaCl, 10 KCl, 5 $MgCl_2$, 0.02 $CaCl_2$, 1.2 $KH_2PO_4$, 50 Taurin, 5 MOPS, 10 BDM, 20 glucose, pH 7.2) with collagenase 1 and protease (0.04%). After 45 min, the supernatant was discarded, and the

remaining tissue poured into the flask. Thereafter, a second digestion step with Tyrode solution with collagenase 1 (without protease) was performed for 20-35 min. The cells were disaggregated using a Pasteur pipette with a wide tip opening and subsequently centrifuged with low g for 10 min. The pellet was resuspended in storage solution containing in mmol/L: 30 KCl, 10 $KH_2PO_4$, 1 $MgCl_2$, 10 HEPES, 11 glucose, 20 taurine, 70 glutamic acid, 20 BDM, 2% BCS, pH 7.4, at room temperature. After isolating cells, Ca was added stepwise to 2 mmol/L and the cell solution was plated onto laminin-coated chambers. For measurements, elongated cells with cross striations were selected, modified according to Neef [9].

### Confocal Ca measurements

Ca sparks were assessed via confocal microscopy using the Ca dye fluo-4 acetoxymethylester. Isolated myocytes were loaded with 10 µmol/L fluo-4 acetoxymethylester (Molecular Probes; for 12 min at room temperature) and mounted on an inverted laser scanning confocal microscope (Zeiss LSM 7). Regular myocyte contraction was elicited by electrical field stimulation (1 Hz) during superfusion with Tyrode solution containing in mmol/L: 140 NaCl, 4 KCl, 5 HEPES, 1 $MgCl_2$, 10 Glucose, 2 $CaCl_2$ (pH 7.4). For measurements of Ca sparks, line scans (512 pixel of 0.075 µm size, 1309 lines per second, 10000 lines per scan, 488 nm excitation, 505 nm long pass emission filter) were acquired immediately after stop of electrical field stimulation. Ca spark characteristics were analyzed using Image J (Sparkmaster plugin (2)). Ca spark frequency (CaSpF) was measured as number of sparks per cell volume and time (100 $\mu m^{-1} s^{-1}$). Ca spark width and duration were taken from the full-width-half-maximum (FWHM) and full-duration-half-maximum (FDHM), respectively. SR Ca leak was calculated as Ca spark frequency x Ca spark amplitude ($F/F_0$) x Ca spark width (full width at half maximum) x Ca spark duration (full duration at half maximum).

For measurement of SR Ca content, myocytes were rapidly exposed to external caffeine (10 mmol/L).

To investigate the influence of CaMKII inhibition on Ca sparks and Caffeine-induced transients we incubated cardiomyocytes for 30 min to CaMKII-Inhibitor autocamtide-2 related autoinhibitory peptide (AIP, 2 µmol/L). For control, the equal amounts of the solvent DMSO (0.1%) were added.

### Western Blot Analysis

RA biopsies were homogenized in protease-inhibitor (Roche) and Tris buffer (in mmol/L: Tris-HCl 20, NaCl 200, NaF 20, $Na_3VO_4$ 1, DTT 1, 1% Triton X-100, pH 7.4). After denaturation (30 min at 37°C in 1% β-mercaptoethanol), proteins were separated on 8% (ox-CaMKII, CaMKII) or 12% (HDAC4, CaMKII-HDAC4) SDS-polyacrylamide gels, then transferred to a nitrocellulose membrane (or PDVF membrane) and incubated with primary antibodies: rabbit

polyclonal anti-CaMKII (1:12000, D.M. Bers' laboratory [10], rabbit polyclonal anti-HDAC4 (1:5000, Santa Cruz), and mouse monoclonal anti-GAPDH (1:50000, BIOTREND) at 4˚C overnight. Secondary antibodies were HRP-conjugated donkey anti-rabbit and sheep anti-mouse IgG (1:10000, GE Healthcare) that were incubated for 1 h at room temperature. For chemiluminescent detection, ImmobilonTM Western Chemiluminescent HRP Substrate (Millipore) was used. For detection of oxidized CaMKII, an immune serum directed against oxizided M281/M282 (ox-CaMKII) was used (polyclonal rabbit, 1:15000, M.E. Anderson's laboratory [11,12]. To avoid unspecific reduction of oxidized CaMKII, β-mercaptoethanol was omitted during the denaturation process (5 min at 95°C). Sometimes, the size of the RA biopsie was too small to allow for simultaneous cell isolation and tissue homogenization. This resulted in a reduced data set for protein analysis compared to the functional data set.

**CaMKII activity assay**

A highly specific CaMKII-activity was used to test CaMKII activity as described previously [13]. Briefly, CaMKII was pulled from homogenized samples using an HDAC4-GST fusion protein. Washing steps with high sodium phosphate-buffered saline (PBS) ensured that only active CaMKII is bound to the HDAC4-fragment (as its binding energy is considerably greater). The activity is analyzed for each pulldown experiment and normalized to the input bait HDAC4-GST in the probe. This intrinsic activity of CaMKII was then normalized to CaMKII expression from the same samples, which was obtained in Western blots and normalized to GAPDH. This normalization yields the specific CaMKII activity referenced in the manuscript.

**Statistical Analysis**

Continuous data are expressed as mean ± standard deviation (SD) and were compared between groups by applying Students t-test. Categorical data are presented as frequencies with percentages and were compared by using the Chi-Square test. For experiments with the CaMKII inhibitor AIP, two-way RM ANOVA (mixed effects model) was used to simultaneously test for differences in patient group and drug treatment.

Simple and multiple linear regression models were used to assess the influence of AHI and additional confounding variables on CaSpF. Confounders were the clinically plausible demographic parameters gender, age, body-mass-index (BMI), N-terminal pro-brain natriuretic peptide (NT-pro BNP) and potential cardiovascular risk factors. Results are presented as regression coefficient (B) with 95% confidence intervals. The coefficient of determination ($R^2$) is also reported. Linear regression assumptions were examined using leverage and Cook's D plots to identify outliers, QQ-plots and distribution of studentized residuals to check normality of the residuals, studentized residuals vs. fitted values to check the constant variance assumption, variance inflation factor to check multi-collinearity and component-residual plots

to check linearity assumptions. A p value ≤0.05 was considered as statistically significant. Statistical analyses were performed with GraphPad Prism 8 (La Jolla California USA) and software package SPSS 22.0 (IBM SPSS Statistics, Armonk, New York, USA).

## Supplemental references

1. Chen, H.; Lowe, A.A.; Bai, Y.; Hamilton, P.; Fleetham, J.A.; Almeida, F.R. Evaluation of a portable recording device (ApneaLink) for case selection of obstructive sleep apnea. *Sleep & breathing = Schlaf & Atmung* **2009**, *13*, 213-219, doi:10.1007/s11325-008-0232-4.
2. Clark, A.L.; Crabbe, S.; Aziz, A.; Reddy, P.; Greenstone, M. Use of a screening tool for detection of sleep-disordered breathing. *The Journal of laryngology and otology* **2009**, *123*, 746-749, doi:10.1017/s0022215109004794.
3. Erman, M.K.; Stewart, D.; Einhorn, D.; Gordon, N.; Casal, E. Validation of the ApneaLink for the screening of sleep apnea: a novel and simple single-channel recording device. *Journal of clinical sleep medicine : JCSM : official publication of the American Academy of Sleep Medicine* **2007**, *3*, 387-392.
4. Ng, S.S.; Chan, T.O.; To, K.W.; Ngai, J.; Tung, A.; Ko, F.W.; Hui, D.S. Validation of a portable recording device (ApneaLink) for identifying patients with suspected obstructive sleep apnoea syndrome. *Internal medicine journal* **2009**, *39*, 757-762, doi:10.1111/j.1445-5994.2008.01827.x.
5. Ragette, R.; Wang, Y.; Weinreich, G.; Teschler, H. Diagnostic performance of single airflow channel recording (ApneaLink) in home diagnosis of sleep apnea. *Sleep & breathing = Schlaf & Atmung* **2010**, *14*, 109-114, doi:10.1007/s11325-009-0290-2.
6. Wang, Y.; Teschler, T.; Weinreich, G.; Hess, S.; Wessendorf, T.E.; Teschler, H. [Validation of microMESAM as screening device for sleep disordered breathing]. *Pneumologie (Stuttgart, Germany)* **2003**, *57*, 734-740, doi:10.1055/s-2003-812423.
7. Arzt, M.; Woehrle, H.; Oldenburg, O.; Graml, A.; Suling, A.; Erdmann, E.; Teschler, H.; Wegscheider, K. Prevalence and Predictors of Sleep-Disordered Breathing in Patients With Stable Chronic Heart Failure: The SchlaHF Registry. *JACC. Heart failure* **2016**, *4*, 116-125, doi:10.1016/j.jchf.2015.09.014.
8. Voigt, N.; Zhou, X.B.; Dobrev, D. Isolation of human atrial myocytes for simultaneous measurements of Ca2+ transients and membrane currents. *Journal of visualized experiments : JoVE* **2013**, e50235, doi:10.3791/50235.
9. Neef, S.; Dybkova, N.; Sossalla, S.; Ort, K.R.; Fluschnik, N.; Neumann, K.; Seipelt, R.; Schondube, F.A.; Hasenfuss, G.; Maier, L.S. CaMKII-dependent diastolic SR $Ca^{2+}$ leak and elevated diastolic $Ca^{2+}$ levels in right atrial myocardium of patients with atrial fibrillation. *Circ Res* **2010**, *106*, 1134-1144, doi:10.1161/circresaha.109.203836.
10. Ai, X.; Curran, J.W.; Shannon, T.R.; Bers, D.M.; Pogwizd, S.M. Ca2+/calmodulin-dependent protein kinase modulates cardiac ryanodine receptor phosphorylation and sarcoplasmic reticulum Ca2+ leak in heart failure. *Circ Res* **2005**, *97*, 1314-1322.
11. Luo, M.; Guan, X.; Luczak, E.D.; Lang, D.; Kutschke, W.; Gao, Z.; Yang, J.; Glynn, P.; Sossalla, S.; Swaminathan, P.D.; et al. Diabetes increases mortality after myocardial infarction by oxidizing CaMKII. *J Clin Invest* **2013**, *123*, 1262-1274, doi:10.1172/jci65268.
12. Erickson, J.R.; Joiner, M.L.; Guan, X.; Kutschke, W.; Yang, J.; Oddis, C.V.; Bartlett, R.K.; Lowe, J.S.; O'Donnell, S.E.; Aykin-Burns, N.; et al. A dynamic pathway for calcium-independent activation of CaMKII by methionine oxidation. *Cell* **2008**, *133*, 462-474, doi:10.1016/j.cell.2008.02.048.
13. Kreusser, M.M.; Lehmann, L.H.; Keranov, S.; Hoting, M.O.; Oehl, U.; Kohlhaas, M.; Reil, J.C.; Neumann, K.; Schneider, M.D.; Hill, J.A.; et al. Cardiac CaM Kinase II genes delta and gamma contribute to adverse remodeling but redundantly inhibit calcineurin-induced myocardial hypertrophy. *Circulation* **2014**, *130*, 1262-1273, doi:10.1161/circulationaha.114.006185.

# Supplemental tables

## Table S1

Linear regression analyses for oxCaMKII/CaMKII

| | Simple Linear Regression Analysis | | Multiple Linear Regression Analysis | | | |
|---|---|---|---|---|---|---|
| | | | Model I $R^2$ 0.476 | | Model II $R^2$ 0.506 | |
| Variable | **B (95% CI)** | **P value** | **B (95% CI)** | **P value** | **B (95% CI)** | **P value** |
| AHI, /h | 0.043 (0.021; 0.065) | **<0.001** | 0.040 (0.016; 0.064) | **0.002** | 0.042 (0.014; 0.071) | **0.007** |
| ODI, /h | 0.036 (0.014; 0.058) | **0.003** | | | | |
| $MinO_2$, % | 0.004 (-0.026; 0.034) | 0.808 | | | | |
| $O_2$ below 90%, % of TRT | -0.001 (-0.016; 0.014) | 0.899 | | | | |
| Age/10, years | 0.024 (-0.259; 0.308) | 0.861 | 0.036 (-0.207; 0.279) | 0.759 | 0.089 (-0.246; 0.417) | 0.588 |
| Male gender | 0.677 (-0.680; 2.034) | 0.311 | 0.119 (-1.077; 1.314) | 0.837 | 0.089 (-1.334; 1.513) | 0.895 |
| Body-mass index, kg/m² | -0.042 (-0.112; 0.027) | 0.220 | -0.027 (-0.092; 0.038) | 0.400 | -0.033 (-0.112; 0.047) | 0.398 |
| NT-pro BNP/1000, pg/ml | 0.018 (-0.031; 0.066) | 0.457 | | | -0.002 (-0.049; 0.047) | 0.923 |
| Diabetes | -0.266 (-0.849; 0.317) | 0.354 | | | -0.161 (-0.772; 0.450) | 0.581 |
| AF | 0.367 (-0.197; 0.931) | 0.190 | | | -0.181 (-0.899; 0.536) | 0.597 |
| ACEi/ARB therapy | -0.242 (-0.982; 0.499) | 0.505 | | | -0.248 (-0.571; 1.066) | 0.527 |
| Beta blocker therapy | -0.061 (-0.748; 0.627) | 0.857 | | | | |
| Valve replacement | 0.059 (-0.586; 0.705) | 0.850 | | | | |
| LVEF < 50% | 0.060 (-0.550; 0.670) | 0.834 | | | | |
| Dilated left atrium | -0.020 (-0.690; 0.650) | 0.948 | | | | |

Model I accounts for age, male gender and BMI. Model II accounts for age, male gender, BMI, NT-pro BNP, diabetes, and AF, and ACEi/ARB therapy.
Abbreviations: AHI: apnea-hypopnea index, ODI: oxygen desaturation index, $MinO_2$: Minimal oxygen saturation, $O_2$ below 90%,: oxygen saturation below 90%, TRT: total recording time, NT-pro BNP: N-terminal pro-brain natriuretic peptide; AF: atrial fibrillation, LVEF: left ventricular ejection fraction, ACEi/ARB: ACE inhibitor/angiotensin-receptor blocker therapy

## Table S2

Linear regression analyses between obstructive apnea index and calcium spark frequency

| | Simple Linear Regression Analysis | | Multiple Linear Regression Analysis Model I $R^2$ 0.308 | |
|---|---|---|---|---|
| Variable | **B (95% CI)** | **P value** | **B (95% CI)** | **P value** |
| Obstructive apnea index, /h | 0.131 (0.042; 0.220) | **0.005** | 0.118 (0.028; 0.209) | **0.012** |
| Age/10, years | 0.04 (-0.291; 0.370) | 0.810 | 0.009 (-0.293; 0.310) | 0.954 |
| Male gender | 1.031 (0.153; 1.909) | **0.023** | 0.804 (-0.052; 1.660) | 0.065 |
| Body-mass index, kg/m² | 0.029 (-0.042; 0.100) | 0.417 | 0.047 (-0.019; 0.113) | 0.156 |

Model I accounts for age, male gender and BMI.

## Table S3

Linear regression analyses between central apnea index and calcium spark frequency

| | Simple Linear Regression Analysis | | Multiple Linear Regression Analysis Model I $R^2$ 0.369 | |
|---|---|---|---|---|
| Variable | **B (95% CI)** | **P value** | **B (95% CI)** | **P value** |
| Central apnea index, /h | 0.064 (0.026; 0.102) | **0.001** | 0.060 (0.023; 0.096) | **0.002** |
| Age/10, years | 0.04 (-0.291; 0.370) | 0.810 | 0.044 (-0.244; 0.331) | 0.758 |
| Male gender | 1.031 (0.153; 1.909) | **0.023** | 0.936 (0.140; 1.732) | **0.023** |
| Body-mass index, kg/m² | 0.029 (-0.042; 0.100) | 0.417 | 0.036 (-0.026; 0.098) | 0.248 |

Model I accounts for age, male gender and BMI.

## Table S4

Linear regression analyses between Cheyne-Stokes Respiration and calcium spark frequency

| | **Simple Linear Regression Analysis** | | **Multiple Linear Regression Analysis Model I $R^2$ 0.245** | |
|---|---|---|---|---|
| Variable | **B (95% CI)** | **P value** | **B (95% CI)** | **P value** |
| Cheyne-Stokes respiration, /10, % of TRT | 0.324 (0.015; 0.633) | **0.040** | 0.291 (-0.180; 0.600) | **0.064** |
| Age/10, years | 0.04 (-0.291; 0.370) | 0.810 | -0.043 (-0.367; 0.281) | 0.788 |
| Male gender | 1.031 (0.153; 1.909) | **0.023** | 0.980 (0.107; 1.852) | **0.029** |
| Body-mass index, kg/m² | 0.029 (-0.042; 0.100) | 0.417 | 0.037 (-0.031; 0.105) | 0.275 |

Model I accounts for age, male gender and BMI. Abbrevation: TRT: total recording time

# Figure S1

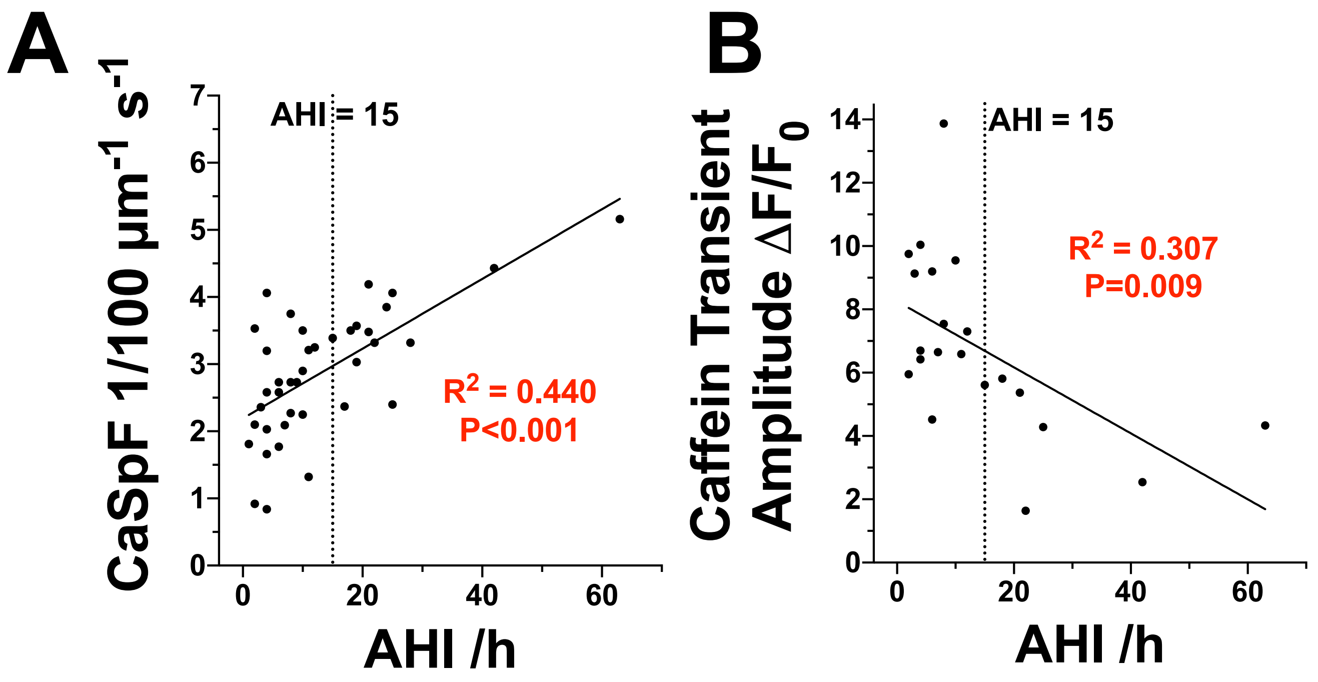


**Figure S1. CaSpF and caffeine-transient amplitude correlated with the severity of SDB.** Scatter plots for the correlation of AHI and CaSpF (A) or caffeine transient amplitude (B), respectively, are shown. CaSpF correlated significantly with AHI (B [95%CI]: 0.05 [0.03;0.07], n=39, $p<0.05$). In accordance, there was a significant negative correlation between AHI and caffeine-transient amplitude (B [95%CI]: -1.04 [-1.79;-0.29], n=21, $p<0.05$).

# Figure S2

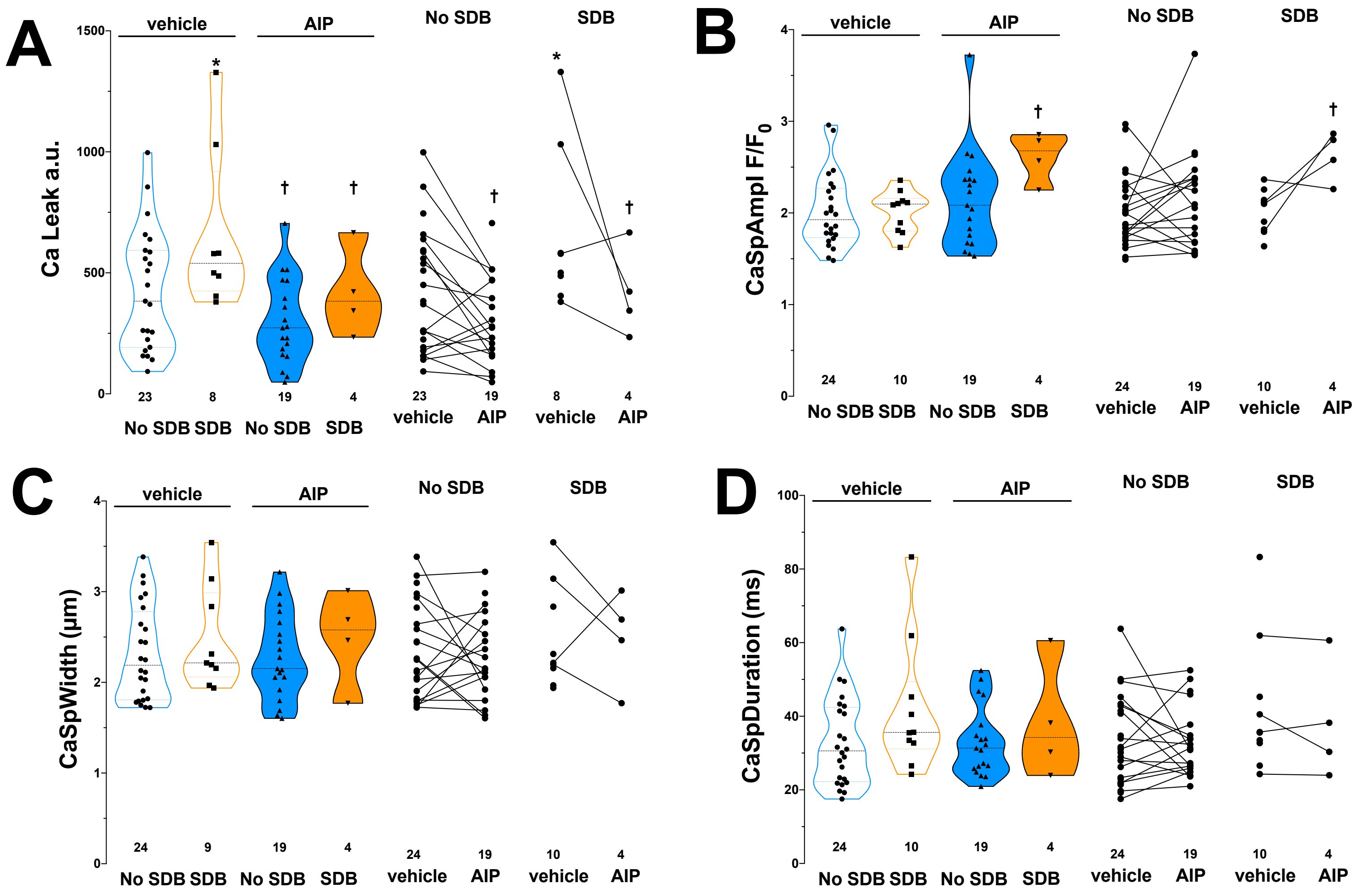


**Figure S2. Patients with SDB showed increased diastolic Ca leak.** A) Mean data for diastolic Ca leak (calculated CaSpF x CaSpAmpl x CaSpDuration x CaSpWidth) is shown as violin plot (left panel) or spaghetti plot (right panel). Compared to control patients (no SDB, mean AHI 3/h), the diastolic Ca leak was significantly enhanced in patients with SDB (mean AHI 28/h). Importantly, this increase in CaSpF could be blocked with the selective CaMKII inhibitor autocamtide-2 related autoinhibitory peptide (AIP). * - P<0.05 vs. No SDB, † - P<0.05 vs. vehicle (two-way RM ANOVA, mixed effects model). B-D) Mean data for Ca spark characteristics Ca spark amplitude (CaSpAmpl, B), Ca spark width at half maximum (CaSpWidth, C), and Ca spark duration at half maximum (CaSpDuration, D). For each, violin plots are shown on left panels and corresponding spaghetti plots on right panels. These characteristics did not differ significantly between patients without or with SDB.

# Figure S3

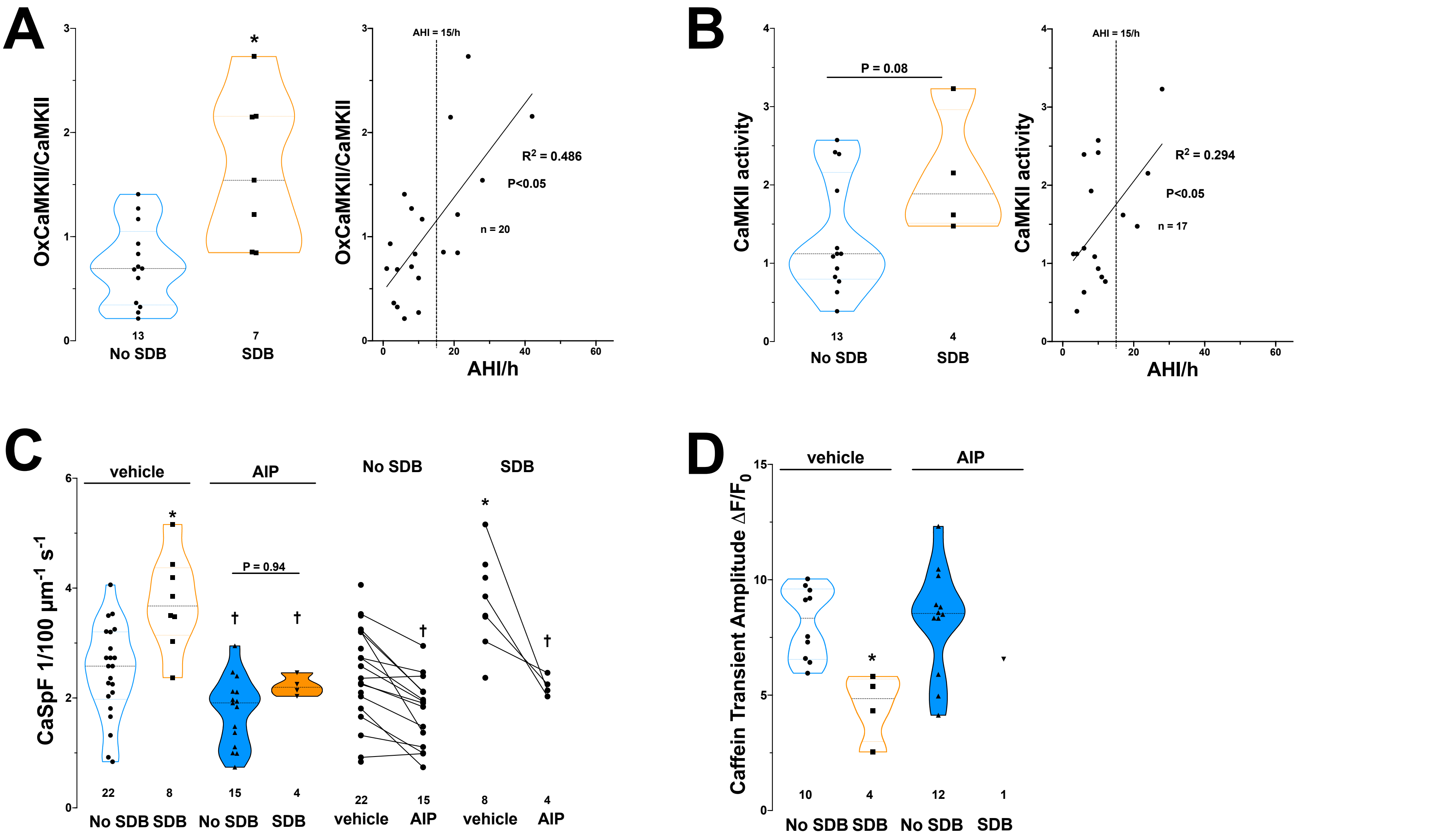


**Figure S3. SDB-dependent atrial remodeling was independent from AF.** Only data from patients with sinus rhythm is shown. A) Oxidized CaMKII/CaMKII levels were increased in patients with SDB. Right panel: scatter plot for the correlation of AHI and oxCaMKII/CaMKII. Significant linear regression is shown as line plot. B) CaMKII activity levels (specific HDAC4 pulldown) are increased in patients with SDB. Right panel: scatter plot for the correlation of AHI and CaMKII-activity. Linear regression analysis is shown as line plot. * - P<0.05 vs. no SDB (t-test). C) Mean data for CaSpF is shown as violin plot (left panel) or spaghetti plot (right panel). Compared to control patients (no SDB), the frequency of Ca sparks was significantly enhanced in patients with SDB. Importantly, this increase in CaSpF could be blocked with the selective CaMKII inhibitor autocamtide-2 related autoinhibitory peptide (AIP). D) Mean data for caffeine-induced Ca transient amplitude. Compared to control patients (no SDB), the caffeine-transient amplitude was significantly lower in patients with SDB. * - P<0.05 vs. no SDB, † - P<0.05 vs. vehicle (two-way RM ANOVA, mixed effects model).
